# Epidemiology of SARS‐CoV‐2 infection and SARS‐CoV‐2 positive hospital admissions among children in South Africa

**DOI:** 10.1111/irv.12916

**Published:** 2021-11-18

**Authors:** Tendesayi Kufa, Waasila Jassat, Cheryl Cohen, Stefano Tempia, Maureen Masha, Nicole Wolter, Sibongile Walaza, Anne von Gottburg, Nelesh P. Govender, Gillian Hunt, Andronica Moipone Shonhiwa, Joy Ebonwu, Genevie Ntshoe, Wellington Maruma, Poncho Bapela, Nomathamsanqa Ndhlovu, Hlengani Mathema, Motshabi Modise, Liliwe Shuping, Pinky N. Manana, David Moore, Ziyaad Dangor, Charl Verwey, Shabir A. Madhi, Haroon Saloojee, Heather J. Zar, Lucille Blumberg

**Affiliations:** ^1^ National Institute for Communicable Diseases National Health Laboratory Services Johannesburg South Africa; ^2^ School of Public Health University of the Witwatersrand Johannesburg South Africa; ^3^ Influenza Division, National Center for Immunization and Respiratory Diseases US Centers for Disease Control and Prevention Atlanta Georgia USA; ^4^ MassGenics Duluth Georgia USA; ^5^ School of Pathology University of the Witwatersrand Johannesburg South Africa; ^6^ School of Health Systems and Public Health, Faculty of Health Sciences University of Pretoria Pretoria South Africa; ^7^ Department of Pediatrics and Child Health University of the Witwatersrand Johannesburg South Africa; ^8^ South African Medical Research Council: Vaccines and Infectious Diseases Analytical Research Unit (VIDA), Faculty of Health Science Johannesburg University of the Witwatersrand Johannesburg South Africa; ^9^ Department of Science/National Research Foundation: Vaccine Preventable Diseases, Faculty of Health Science Johannesburg University of the Witwatersrand Johannesburg South Africa; ^10^ Dept of Paediatrics and Child Health, Red Cross Children's Hospital, and SA‐MRC Unit on Child and Adolescent Health University of Cape Town Cape Town South Africa

**Keywords:** adolescents, children, COVID‐19, incidence, mortality, testing rate

## Abstract

**Introduction:**

We describe epidemiology and outcomes of confirmed SARS‐CoV‐2 infection and positive admissions among children <18 years in South Africa, an upper‐middle income setting with high inequality.

**Methods:**

Laboratory and hospital COVID‐19 surveillance data, 28 January ‐ 19 September 2020 was used. Testing rates were calculated as number of tested for SARS‐CoV‐2 divided by population at risk; test positivity rates were calculated as positive tests divided by total number of tests. In‐hospital case fatality ratio (CFR) was calculated based on hospitalized positive admissions with outcome data who died in‐hospital and whose death was judged SARS‐CoV‐2 related by attending physician.

**Findings:**

315 570 children aged <18 years were tested for SARS‐CoV‐2; representing 8.9% of all 3 548 738 tests and 1.6% of all children in the country. Of children tested, 46 137 (14.6%) were positive. Children made up 2.9% (n = 2007) of all SARS‐CoV‐2 positive admissions to sentinel hospitals. Among children, 47 died (2.6% case‐fatality). In‐hospital deaths were associated with male sex [adjusted odds ratio (aOR) 2.18 (95% confidence intervals [CI] 1.08–4.40)] vs female; age <1 year [aOR 4.11 (95% CI 1.08–15.54)], age 10–14 years [aOR 4.20 (95% CI1.07–16.44)], age 15–17 years [aOR 4.86 (95% 1.28–18.51)] vs age 1–4 years; admission to a public hospital [aOR 5.07(95% 2.01–12.76)] vs private hospital and ≥1 underlying conditions [aOR 12.09 (95% CI 4.19–34.89)] vs none.

**Conclusions:**

Children with underlying conditions were at greater risk of severe SARS‐CoV‐2 outcomes. Children > 10 years, those in certain provinces and those with underlying conditions should be considered for increased testing and vaccination.

## 1 BACKGROUND

Since its emergence in Wuhan, China, in December 2019, the novel SARS‐CoV‐2, the virus that causes coronavirus disease 2019 (COVID‐19) has culminated in a global pandemic with global cases and deaths continuing to climb.^1^ By September 19, 2020, the cut‐off date for this report, 30.6 million cases of COVID‐19 and 95, 000‐related deaths had been reported to the World Health Organization (WHO),^1^ and children aged <18 years were estimated to have contributed to 8.5% of COVID‐19 cases globally.^2^, ^3^, ^4^ However, the proportion of cases in children varied by country and was as high as 11% in the United States, 23% in Paraguay, and 15% in Brazil.^5^

Several factors might contribute to the lower reported incidence of COVID‐19 among children: SARS‐CoV‐2 infection is more likely to be asymptomatic or cause milder symptoms in children (80%) than in adults (40–60%), and children may be less likely to receive medical care or be tested.^2^, ^6^, ^7^, ^8^, ^9^, ^10^ Furthermore, some data suggest children under 10 years of age might be 48% less susceptible to infection following exposure compared to adults.^10^ Therefore, the testing rates could be lower in that population.^11^, ^12^

Published data from the United States suggest rates of admission to hospital and intensive care units (ICUs) have been lower among children with COVID‐19 (cumulative hospitalization rates eight cases per 100,000 children <18 years) than adults (164.5 cases per 100,000 adults).^13^, ^14^ Systematic reviews of studies published up to May 2020 reported pediatric case fatality ratio (CFR) ranging between 0% (in two studies) and 0.2% across their respective included studies.^15^, ^16^, ^17^, ^18^, ^19^ In a study of 114,000 SARS‐CoV‐2 positive deaths in the United States, children aged <18 years made up <0.3% of all deaths.^20^ Subsequent studies of children hospitalized with COVID‐19 have described greater risk of severe disease especially among children with obesity and other underlying medical conditions compared to those without these conditions.^13^, ^21^, ^22^

There is a paucity of studies describing the epidemiology and clinical features of COVID‐19 among children in Africa. Of published studies, a relatively small number of children were studied, ranging from 34–1,439.^23^, ^24^, ^25^ Findings from China,^7^ Europe,^15^ and North America,^19^ where the majority of studies of COVID‐19 in children were conducted, may not be generalizable to countries such as South Africa where conditions such as malnutrition, childhood obesity, tuberculosis, HIV infection, or HIV exposure among children are more prevalent and background child mortality rates from other bacterial and viral infections are higher than other countries.^26^, ^27^ Additionally, sub‐optimal sanitation, overcrowding, and limited access to health care likely reduce the efficacy of non‐pharmacological interventions against COVID‐19. We describe the epidemiology of SARS‐CoV‐2 infection and hospitalization among children aged <18 years in South Africa. Specifically, we describe age‐specific population level testing rates, incidence of laboratory‐confirmed SARS‐CoV‐2 infection, and the clinical characteristics and outcomes of children admitted with COVID‐19 at sentinel hospitals. We also identify modifiable factors associated with in‐hospital deaths to guide interventions for this population.

## 2 METHODS

### 2.1 Setting

South Africa is an upper middle‐income country with high income inequality—GINI coefficient of 0.65 at national level.^28^ The country had an estimated 2020 mid‐year population of 59.6 million, of which 33.5% (an estimated 20 million) were children <18 years old.^29^ In 2019, the country's under five mortality rate was 34 per 1,000 live births compared to 2 per 1,000 in the Nordic countries, 6 per 1,000 in the United States, and 117 per 1000 in Nigeria and Somalia.^29^, ^30^ The country is divided administratively into nine provinces in which there are wide variations in income and healthcare access and quality. The majority of the population lives in low‐income settings characterized by high levels of unemployment and limited access to medical insurance and medical care. South Africa started real time reverse transcription polymerase chain reaction (rRT‐PCR) testing for SARS‐CoV‐2 infection on January 28, 2020, and the first case of SARS‐CoV‐2 infection was reported on March 5, 2020. As part of non‐pharmaceutical interventions to curb the spread of the epidemic, schools were closed on March 18, 2020, and measures restricting non‐essential travel and trade were introduced on March 27, 2020. Restrictions were gradually eased starting May 1, 2020, paradoxically as the epidemic started to surge, with phased reopening of schools from June 8, 2020, with all children returning to school by September 1, 2020.

### 2.2 Data sources and collection procedures

SARS‐CoV‐2 rRT‐PCR results were reported by both public and private laboratories to a surveillance system coordinated by the National Institute for Communicable Diseases (NICD). Limited demographic and epidemiological data such as age, sex, and contact information were obtained at the time of specimen collection. People meeting the South African National Department of Health (NDOH) case definition for persons under investigation (PUI) were tested. In March 2020, a PUI was defined as a person, regardless of age, with acute onset of fever >38.5°C with one or more of the following: cough, fever, or sore throat and contact with a known case of COVID‐19.^31^ This definition was revised several times over the reporting period. For example on June 1, 2020, the guidance around which individuals could be tested by SARS‐CoV‐2 rRT‐PCR was changed to restrict testing to those with symptoms, those who needed admission, and those with underlying conditions.^31^ For hospitalized people, data were collected at admission, during hospitalization, and at discharge using the DATCOV system, a prospective surveillance program for sentinel hospitals. The DATCOV system was introduced in phases starting April 2020.^32^ Healthcare workers treating COVID‐19 patients of all ages reported demographic characteristics, clinical signs and symptoms, treatment provided, presence of underlying medical conditions, and outcomes among patients who had positive SARS‐CoV‐2 rRT‐PCR tests and were admitted to participating sentinel hospitals (including public [government owned and operated] and private [individual or non‐government entity owned and operated] facilities), using a structured electronic form. In addition to direct data captured by hospital staff, some sentinel hospitals exported data from COVID‐19 admissions into the DATCOV system. The number of reporting, sentinel hospitals expanded during the study period. By September 19, 2020, there were 513 hospitals (269 public and 244 private hospitals) reporting COVID‐19 admissions on the DATCOV platform. This represented 100% of private sector hospitals and 88% of all public sector hospitals in the country.^33^ Because not all hospitals started reporting admissions at the same time, hospitals which started reporting later in the surveillance period could submit their data retrospectively to include all admissions regardless of age.^32^

### 2.3 Laboratory procedures

Testing for SARS‐CoV‐2 using rRT‐PCR began on January 28, 2020, at the reference laboratory at NICD and was expanded to a national network of private and public laboratories at the beginning of March 2020. The SARS‐CoV‐2 rRT‐PCR testing from public sector facilities was free to the user while testing at private sector facilities required user fees in cash or through health insurance. By September 19, 2020, public sector laboratories accounted for 45.8% of all cumulative tests conducted and 41.4% of all positive tests, despite serving an estimated 80% of the population.^34^ Respiratory specimens, including nasopharyngeal swabs, nasal swabs, oropharyngeal swabs, and occasionally lower respiratory tract specimens (sputum, tracheal aspirate, and broncho‐alveolar lavage) were collected at the discretion of the attending healthcare worker and submitted to testing laboratories. Laboratories used any one of several in‐house and commercial rRT‐PCR assays including the TIB Molbiol LightMix® Modular SARS‐CoV (COVID19) assay (Roche Diagnostics, Basel, Switzerland) and Allplex™ 2019‐nCoV assay (Seegene, Seoul, Republic of Korea) to test for the presence of SARS‐CoV‐2 RNA. Test results were automatically fed into the NICD data warehouse after result confirmation. Patients received their results through a short‐text messaging system (SMS) directly from the laboratory or through the ordering physician.

### 2.4 Data management

Data on SARS‐CoV‐2 rRT‐PCR tests conducted and case notifications were extracted from laboratory information systems, while data on hospitalizations were extracted from the DATCOV platform. Once extracted, duplicate entries by name and date of birth were identified and removed. The hospital dataset was compared with the line list of all laboratory‐confirmed cases in order to exclude admissions that were not laboratory‐confirmed. Data captured in SARS‐CoV‐2 testing database, case line list, and DATCOV database as of September 19, 2020, were extracted on the September 22, 2020, and exported into STATA® 14.2 (Stata Corporation, College Station, Texas, United States) for analysis.

### 2.5 Data analysis

Descriptive statistics were used to determine the age‐specific testing, percent positive and incidence rates, and case fatality ratios (CFRs) comparing children (age <18 years) to adults (age ≥18 years) overall. Among children, descriptive statistics were used to describe testing rate, percent positive proportion, incidence rate, and case fatality ratios by age categories, sex, epidemiology week, and location (province). The main outcomes analyzed were (1) the SARS‐CoV‐2 rRT‐PCR testing rate and percent positive, (2) incidence of laboratory‐confirmed SARS‐CoV‐2 infection, and (3) in‐hospital CFR among SARS‐CoV‐2‐positive admissions. The testing rate (per 100,000 persons) was determined as the number of unique SARS‐CoV‐2 rRT‐PCR tests—excluding repeat tests—divided by the population size based on the 2020 mid‐year population estimates,^29^ stratified by age and sex and epidemiology week. The incidence of laboratory‐confirmed SARS‐CoV‐2 infection was also presented as number of new infections per 100,000 persons. A SARS‐CoV‐2 positive admission was a person admitted to hospital with a confirmed SARS‐CoV‐2 rRT‐PCR positive result regardless of the reason for admission. The CFR among hospitalized patients was determined as the percentage of SARS‐CoV‐2 positive admissions with a documented outcome who died during their stay at a sentinel hospital and whose death was possibly SARS‐CoV‐2 infection‐related as determined by the attending physicians. Having an underlying condition was determined as report of at least one of the following chronic, pre‐existing conditions at admission: asthma or any other chronic respiratory disease, any diabetes, hypertension, chronic kidney disease, a chronic heart disease, any history of malignancy, active or previous tuberculosis, HIV infection, or another chronic illness the children had at admission and coded as “other.” Information on underlying conditions was obtained from medical record review or self‐reported by patient or caregiver.

Univariate and multivariable random effects logistic regression with and without multiple imputation were developed to determine factors independently associated with in‐hospital death. Covariates assessed a priori in the model were age, sex, province, admission at public versus private hospitals, and presence of an underlying medical condition. A random effects model taking into account admission facility was used to account for potential differences in the service population and the quality of care of each facility, whereas chained equation multiple imputation over 10 imputation runs was used to account for missing data on the selected covariates. Incomplete variables included in the imputation chain were ethnicity, hospital admission month, and comorbidities such as HIV infection, tuberculosis (TB) infection, hypertension, diabetes, malignancy, reported obesity, asthma, chronic pulmonary disease, cardiac disease, and renal disease. Complete variables included in the imputation model were sex, province, whether a child was admitted to a private or public hospitals, and the in‐hospital outcome. Analysis of admissions was limited to the first admission for COVID‐19. Because of low burden of most underlying conditions, reported underlying conditions were grouped together as absence or presence of any non‐communicable comorbid condition(s). For the multivariable model, we assessed all variables that were statistically significant at *p* < 0.2 on univariate analysis and dropped non‐significant factors (*p* ≥ 0.05) using manual backward elimination.

### 2.6 Ethical considerations

The NICD has ethical clearance for essential communicable diseases surveillance and outbreak response investigation activities from the University of the Witwatersrand's Human Research Ethics Committee (Medical) (M160667). This activity was reviewed by CDC and was conducted consistent with applicable federal law and CDC policy.* All personal and identifying information were removed, and case records were uniquely assigned to a system‐generated record identifier.

## 3 RESULTS

### 3.1 Testing rate and SARS‐CoV‐2 rRT‐PCR percentage test positive

**FIGURE 1:**
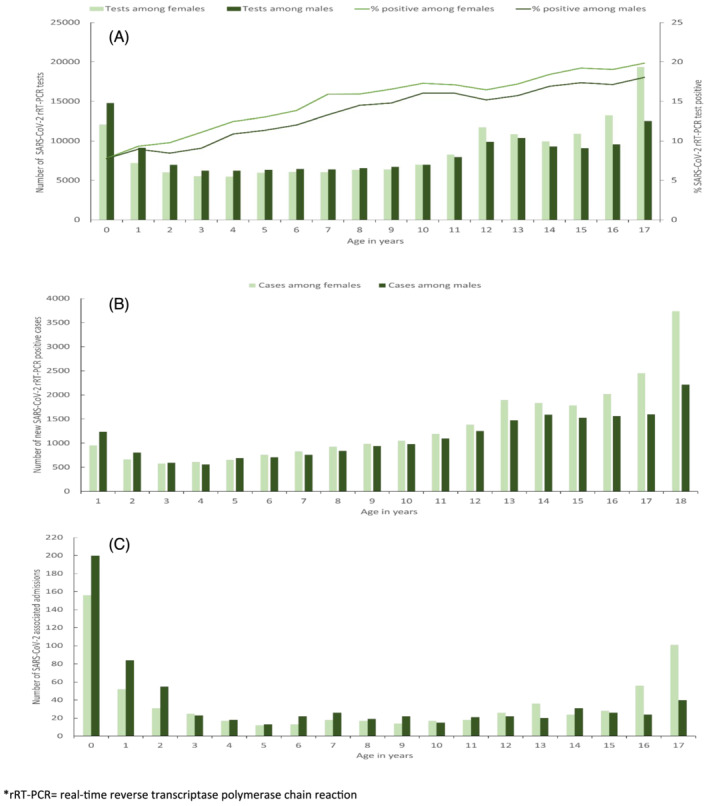
SARS‐CoV2 rRT‐PCR: (A) number of tests and percent positive, (B) number of new positive cases, and (C) number of associated‐ hospital admissions among children <18 years, by age and sex, South Africa, March 1, 2020, to September 19, 2020. rRT‐PCR, real‐time reverse transcriptase polymerase chain reaction

**FIGURE 2:**
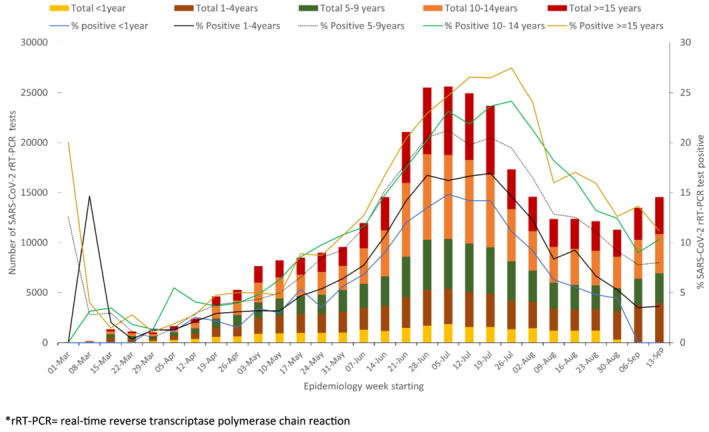
Weekly number of SARS‐CoV‐2 rRT‐PCR8^*^ tests and percent positive among children <18 years by age, South Africa, March 1, 2020 to September 19, 2020. rRT‐PCR, real‐time reverse transcriptase polymerase chain reaction

From January 28 to September 19, 2020, 3,548,738 unique SARS‐CoV‐2 rRT‐PCR tests were performed across all age groups (5,952 tests per 100,000 persons). Of these, 315,570 (8.9%) were performed on children aged <18 years, a rate of 1581 tests per 100,000 persons, which was fivefold lower than among adults who had a rate of 8,153 tests per 100,000 persons. The number of tests conducted varied by province (Table 1). A SARS‐CoV‐2 rRT‐PCR test was positive in 46,137/313,584 valid tests (14.6%) among children compared to 587,460/3,186,450 tests (18.4%) among adults. Among children, the percentage of positive SARS‐CoV‐2 rRT‐PCR tests increased with age from 7.8% (2,120/27,253) among those aged <1 year to 19.1% (6,155/32,147) in those aged 17 years (Figure 1A). The weekly testing rate among children increased from <1 per 100,000 persons in epidemiology week March 1–7, 2020, peaking at 128 per 100,000 persons in week July 5–12, 2020, then declining steadily to <74 per 100,000 persons during weeks August 2 to September 19, 2020. The percentage of SARS‐CoV‐2 rRT‐PCR test positivity among children varied with age categories <18 years, but overall increased from a low of 1.1% in weeks March 29 to April 4, 2020, peaking at 22.3% in week July 19–25, 2020, before declining to 8.3% in week August 16–22, 2020 (Figure 2).

**TABLE 1:** Distribution of total population, SARS‐CoV‐2 rRT‐ PCR tests, positive cases, and SARS‐CoV‐2 positive hospital admissions among children <18 years by province, South Africa, March 1, 2020, to September 19, 2020

Province	Total population^a^	Population aged <18 years in the province ^b^	Population of children in province as % of total population^c^	Population of children in province as % of all children in country^d^	SARS‐CoV‐2 rRT‐PCR tests done on children *n* (%)^e^	SARS‐CoV‐2 rRT‐PCR testing rate per 100,000^f^	SARS‐CoV‐2 rRT‐PCR percent positive (%)^g^	SARS‐CoV‐2 rRT‐PCR positive cases reported *n* (%)^h^	Incidence per 100,000^i^	SARS‐CoV‐2 admissions n (%)^j^
Eastern Cape	6,734,001	2,598,744	38.6	13.0	37,651 (11.9)	1,448.8	18.8	7,422 (16.3)	285.6	225 (11.2)
Free State	2 ,928,903	987,049	33.7	4.9	24,534 (7.8)	2,485.6	16.9	4,026 (8.8)	407.9	137 (6.8)
Gauteng	15,488,137	4,268,374	27.6	21.4	90,697 (28.7)	2,124.9	14.6	12,709 (27.9)	297.7	413 (20.6)
KwaZulu Natal	11,531,628	4,321,495	37.5	21.6	75,630 (24.0)	1,750.1	13.5	10,023 (22.0)	231.9	360 (17.9)
Limpopo	5,852,553	2,310,964	39.5	11.6	11,315 (3.6)	489.6	9.9	1,264 (2.8)	54.7	58 (2.9)
Mpumalanga	4,679,786	1,621,363	34.6	8.1	13,991 (4.4)	862.9	15.2	1,986 (4.4)	122.5	60 (3.0)
North West	4,108,816	1,404,681	34.2	7.0	8,199 (2.6)	583.7	16.5	1,734 (3.8)	123.4	81 (4.0)
Northern Cape	1,292,786	440,893	34.1	2.2	7,642 (2.4)	1,733.3	17.2	1,601 (3.5)	363.1	44 (2.2)
Western Cape	7,005,741	2,012,667	28.7	10.1	43,058 (13.6)	2,139.4	11.8	4,844 (10.6)	240.7	629 (31.3)
Total	59,622,350	19,966,230	33.5	100.0	315,570^k^ (100.0)	1,580.5	14.6	45,609 (100.0)	228.4	2007 (100.0)

Abbreviation: rRT‐ PCR, real‐time reverse transcriptase polymerase chain reaction.

^a^ Total population of South Africa according to 2020 Statistics South Africa mid‐year population (available from http://www.statssa.gov.za/?p=13453).

^b^ Population of children <18 years according to 2020 Statistics South Africa mid‐year population (available from http://www.statssa.gov.za/?p=13453).

^c^ Proportion of population represented by children <18 years (calculated as 2 as a fraction of 1).

^d^ Proportion of the national population of children found in each province according to 2020 Statistics South Africa mid‐year population (available from http://www.statssa.gov.za/?p=13453).

^e^ Number of SARS‐CoV‐2 tests conducted among children in each province and as proportion of total number of tests conducted among children nationally.

^f^ Testing rate among children, determined as number of tests^5^ divided by population aged <18 years^2^.

^g^ Proportion of valid childhood tests which were positive.

^h^ SARS‐Co‐V‐2 cases among children <18 years reported from each province during the surveillance period and as percentage total number of cases reported in the surveillance period.

^i^ Number of cases per 100,000 population. Determined as number of SARS‐CoV‐2 cases reported^7^ divided by population aged <18 years.^2^

^j^ SARS‐Co‐V‐2‐associated admissions among children <18 years reported from each province during the surveillance period and as percentage total number of associated admissions among children <18 years.

^k^ Included 2,583 (0.9%) for whom province was unknown.

### 3.2 Incidence of laboratory confirmed SARS‐CoV‐2 infection

Overall, 662,343 (18.7%) people had tested positive on SARS‐CoV‐2 rRT‐PCR and were reported to the NICD by September 19, 2020. Of the 640,449 (96.7%) who had age data available, 45,609 (7.1%) were children (0–17 years old). Among children testing positive, the median age was 12.4 years (interquartile range [IQR] 7.4–15.7 years), and 20,412 (44.8%) were male (Table S1). Most SARS‐CoV‐2 positive children (85.6%) resided in the country's five most populous provinces—Eastern Cape, Free State, Gauteng, KwaZulu‐Natal, and Western Cape Provinces (Table 1). The overall cumulative incidence risk among children was 228.4 per 100,000 persons, which was sevenfold lower than in adults (incidence 1,615 per 100,000 persons). The cumulative incidence among children aged <2 years old (160.9 per 100,000 persons) was higher in males (173.7 per 100,000 persons) than females (141.5 per 100,000 persons), but lower than in children aged ≥2 years (237.3 per 100,000 persons) (Figure 1B). Nationally, the weekly incidence increased from <1 per 100,000 children during week March 1–7 peaking at 27.5 per 100,000 children in week July 5–11 (Figure S1B).

### 3.3 Characteristics of SARS‐CoV‐2 positive hospital admissions

There were 70,622 new SARS‐CoV‐2 rRT‐PCR positive admissions captured on DATCOV during the surveillance period. Of these, 185 (0.3%) were excluded for missing age data, false positive results or because patients were from long‐term care facilities. Of those with age data available (*N* = 70,437), only 2,007 (2.9%) were children. The number of SARS‐CoV‐2 rRT‐PCR positive admissions varied by age and province (Tables 1 and S2). The median age of hospitalized children was 6.8 years (IQR 1.1–14.4 years) of whom 1,004 (50.0%) were male. SARS‐CoV‐2 rRT‐PCR admissions among children were highest in infants (age <1 year) and children 13–17 years but lowest among those aged 3 to 12 years (Figure 1C). Admissions increased from one in week March 1–7, 2020, to a peak of 172 in week July 12–18, 2020 (Figure S1C).

**FIGURE 3:**
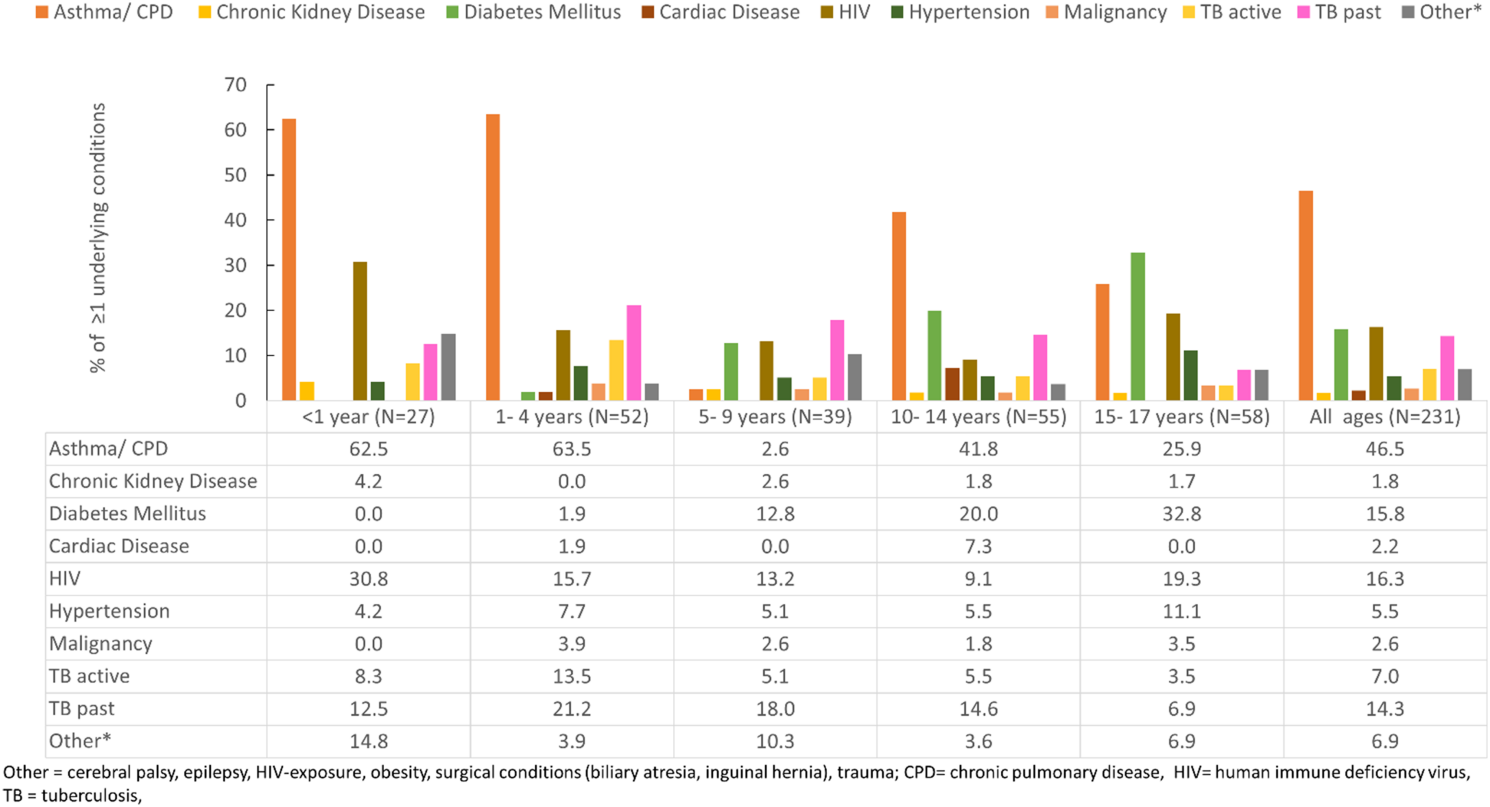
Proportion of SARS‐CoV‐2 associated hospital admissions of children aged <18 years with reported underlying medical conditions among those with ≥1 underlying conditions, by age group, South Africa, March 1, 2020, to September 19, 2020

Tables 2 and S2 describe the clinical characteristics of hospitalized children overall, categorized by specified age categories and by province. Of the 1,426 children with data on underlying medical conditions, 231 (16.1%) had ≥1 documented conditions reported. The most common comorbidities were chronic respiratory (106/231, 46.7%), HIV infection (37/231, 16.3%), and diabetes mellitus (36/231, 15.9%), although the frequency of underlying conditions varied with age category (Figure 3). Data on underlying medical conditions was available for 915/1,119 (81.9%) of children admitted to private sector hospitals versus 511/890 (57.8%) among those admitted to public sector hospitals. However, underlying conditions were more frequently reported among children admitted at public versus private hospitals (152/511 [29.8%] vs. 79/915 [8.6%], respectively, *p* < 0.001).

**TABLE 2:** Characteristics of COVID‐19 associated hospital admissions to sentinel hospitals by children <18 years by age, South Africa, March 1, 2020, to September 19, 2020 (*N* = 2,007)

Variable	<1 year	1–4 years	5–9 years	10–14 years	15–17 years	All children
	(*N* = 469)	(*N* = 449)	(*N* = 267)	(*N* = 366)	(*N* = 456)	(*N* = 2,007)
Age						
Age (median, IQR)	2.7 (0.8–6.5) months	2.2 (1.5–3.3) years	7.6 (6.4–8.8) years	12.9 (11.4–14.0) years	17.0 (16.1–17.6) years	6.8 (1.1–14.4) years
Sex						
Male, *n* (%)	260 (55.4)	255 (56.8)	156 (58.4)	177 (48.4)	156 (34.3)	1,004 (50.0)
Province						
Eastern Cape	28 (6.0)	32 (7.1)	24 (9.0)	44 (12.0)	97 (21.3)	225 (11.2)
Free State	14 (3.0)	32 (7.1)	15 (5.6)	31 (8.5)	45 (9.9)	137 (6.8)
Gauteng	94 (20.0)	94 (20.9)	63 (23.6)	82 (22.3)	80 (17.5)	413 (20.6)
Kwa‐Zulu Natal	82 (17.5)	70 (15.6)	50 (18.7)	83 (22.7)	75 (16.5)	360 (17.9)
Limpopo	14 (3.0)	5 (1.1)	12 (4.5)	11 (3.0)	16 (3.5)	58 (2.9)
Mpumalanga	13 (2.8)	16 (3.6)	6 (2.3)	8 (2.2)	17 (3.7)	60 (3.0)
North‐West	9 (1.9)	15 (3.3)	8 (3.0)	15 (4.1)	34 (7.5)	81 (4.0)
Northern Cape	5 (1.1)	10 (2.2)	8 (3.0)	14 (3.8)	7 (1.5)	44 (2.2)
Western Cape	210 (45.8)	175 (39.0)	81 (30.3)	78 (21.2)	85 (18.6)	629 (31.3)
Hospital						
Public hospital, *n* (%)	238 (50.8)	171 (38.1)	108 (40.5)	162 (44.3)	211 (46.3)	890 (44.3)
Data available on underlying conditions (yes, *n* [%])	256 (54.6)	307 (68.4)	200 (74.9)	281 (76.8)	382 (83.8)	1,426 (71.1)
≥1 underlying conditions reported, *n* (%)^a^	27 (10.6)	52 (16.9)	39 (19.5)	55 (19.6)	58 (15.2)	231 (16.2)
LOS, days (median, IQR)	4 (2–9)	3 (1–5)	2 (1–6)	3 (1.5–8)	4 (2–7.5)	3 (2–7)
Ever admitted to high care, *n* (%)	28 (6.0)	15 (3.3)	7 (2.6)	20 (5.5)	14 (3.1)	84 (4.2)
Ever admitted ICU, *n* (%)	64 (13.7)	24 (5.4)	18 (6.7)	27 (7.4)	21 (4.6)	154 (7.7)
Ever ventilated, *n* (%)	25 (5.3)	5 (1.1)	10 (3.8)	12 (3.3)	5 (1.0)	57 (2.8)
Discharged alive, *n* (%)^b^	413 (88.1)	409 (91.1)	237 (88.8)	314 (85.8)	396 (86.8)	1,769 (88.1)
Transferred out, *n* (%)	4 (0.9)	3 (0.7)	0 (0.0)	7 (1.9)	2 (0.4)	16 (0.8)
Still admitted, *n* (%)	38 (8.1)	34 (7.6)	25 (9.4)	34 (9.2)	43 (9.4)	174 (8.7)
Died, *n* (%)^b^ ^,^ ^c^	13 (2.8)	3 (0.7)	5 (1.9)	11 (3.0)	15 (3.3)	47 (2.3)

Abbreviations: ICU, intensive care unit, IQR, interquartile range; LOS, length of stay in hospital.

^a^ Denominator is those with data on underlying conditions.

^b^ Only these were included in the analysis for in‐hospitals analysis as they had outcome data available.

^c^ Excluding one death considered unrelated to SARS‐CoV‐2 infection.

### 3.4 In‐hospital case fatality ratio

Among the SARS‐CoV‐2 rRT‐PCR positive childhood admissions (*n* = 2,007), the median length of hospital stay was 3 days (IQR 1–7 days). One hundred and fifty‐four children (7.7%) were admitted into an ICU, of whom 57 were ventilated (2.8%). Most children (1,769, 88.1%) were discharged alive, 174 (8.7%) were still hospitalized, 16 (0.8%) were transferred to other hospitals, and 48 (2.4%) died. Of the 48 in‐hospital deaths, one (0.1%) reportedly died from causes unrelated to SARS‐CoV‐2 infection (hyaline membrane disease and associated prematurity). The in‐hospital CFR was 2.6% (47/1,817), 7.6‐fold lower than that in adults—19.8% (12,536/63,473). Deaths among pediatric SARS‐CoV‐2 positive admissions represented 0.4% of all in‐hospital SARS‐CoV‐2 positive deaths of all ages.

Among the 47 children who died from causes possibly related to SARS‐CoV‐2 infection, 30 (63.8%) were male, and the median age was 11.5 years (IQR 5.6 months to 15.9 years). Close to a third of the children who died were aged <1 year (27.7% vs. 23.4% among all admitted children, *p* = 0.287). As expected, a higher proportion of the children who died had been admitted to the ICU (46.1% vs. 6.1%, *p* < 0.001) or had been ventilated (25.5% vs. 2.3%, <0.001) at some point during admission compared to those discharged. Diabetes (4/15–26.7%) and cardiac disease (3/15–20.0%) were the most frequently reported underlying conditions among the children who died (Table 3). TB and HIV were not independently associated with mortality.

**TABLE 3:** Characteristics of children <18 years with COVID‐19 associated hospital admission who died in‐hospital, South Africa, March 1, 2020, to September 19, 2020 (*N* = 47)^a^

Characteristic	*n* (%)
Age	
Median age (IQR)	11.5 years (5.6 months to 15.9 years)
Age group	
<1 year	13 (27.7)
1–4 years	3 (6.4)
5–9 years	5 (10.6)
10–14 years	11 (23.4)
15–17 years	15 (31.9)
Sex	
Male	30 (63.8)
Province	
Eastern Cape	10 (21.3)
Free State	4 (8.5)
Gauteng	9 (19.2)
KwaZulu‐Natal	8 (17.0)
Western Cape	13 (27.7)
Others^b^	3 (6.3)
Severity of disease	
High care	7 (14.9)
ICU admission	22 (46.1)
Ventilated	12 (25.5)
Underlying conditions^c^	
Data on underlying conditions available	38/47 (80.8)
≥1 Underlying Conditions	15/38 (39.5)
Diabetes	4/15 (26.7)
Cardiac condition	3/15 (20.0)
Chronic kidney disease	2/15 (13.3)
Malignancy	2/15 (13.3)
HIV infection	2/15 (13.3)
Obesity	2/15 (13.3)
Hypertension	1/15 (6.7)
Previous TB infection	1/15 (6.7)
Active TB infection	0
Asthma/Chronic pulmonary disease	0
Other‐ biliary duct atresia, paralytic ileus	2/15 (13.3)

Abbreviations: ICU, intensive care unit; IQR = interquartile range; TB, tuberculosis; HIV, human immunodeficiency virus.

^a^ A SARS‐CoV‐2 associated admission was defined as an individual who had a SARS‐CoV‐2 rRT‐PCR positive result AND was admitted to a sentinel hospital regardless for reason for admission.

^b^ Limpopo and Mpumalanga. There were no deaths in North West and Northern Cape.

^c^ An individual could have more than 1 underlying conditions.

In a multivariable model on imputed data adjusting for age, sex, admission in public or private hospital, and presence of one or more non‐communicable underlying conditions (excluding HIV infection and TB), mortality was independently associated with (1) age <1 year (adjusted odds ratio [aOR] 4.11 [95% CI 1.08–15.54]); 10–14 years (aOR 4.20 [95% CI 1.07–16.44]); 15–17 years (aOR 4.86 [95% CI 1.28–15.81]) compared to age 1–4 years; (2) male sex (aOR 2.18 [95% CI 1.08–4.40]) compared to female; (3) admission at a public hospital (aOR 5.07 [95% CI 2.01–12.76]) compared to a private one, and (4) having ≥1 non‐communicable underlying medical condition (aOR 12.09 [95% CI 4.19–34.89]) compared to having none (Table 4). Similar findings were observed using the same multivariable model on unimputed data, although the model run with unimputed data had a lower odds ratio for the association between mortality and having one or more underlying conditions (Tables S3 and S4). Ethnicity was not associated with mortality in univariable analyses and was therefore not included in multivariable models with imputed or unimputed data.

**TABLE 4:** Factors associated with in‐hospital death among COVID‐19 associated hospital admissions in children <18 years, South Africa, March 1, 2020 to September 19, 2020 (*N* = 1,817)^a^

Variable	CFR (95% CI)	Univariate OR (95% CI)^b^	Multivariate OR (95% CI)^b^
Age in years			
<1 years	3.1 (1.4–4.7)	4.22 (1.18–15.18)	4.11 (1.08–15.54)
1–4 years	0.7 (0.0–1.6)	1.00	1.00
5–9 years	2.1 (0.2–3.9)	2.85 (0.66–12.25)	2.47 (0.53–15.54)
10–14 years	3.4 (1.4–5.3)	4.92 (1.33–18.14)	4.20 (1.07–16.44)
15–17 years	3.6 (1.8–5.5)	5.65 (1.58–20.28)	4.86 (1.28–18.51)
Male			
No	1.9 (1.0–2.8)	1.00	1.00
Yes	3.3 (2.1–4.4)	1.70 (0.92–3.15)	2.18 (1.08–4.40)
Ethnicity			
White	2.2 (0–5.7)	1.00	–
Black African	2.4 (0–4.9)	1.27 (0.19–8.53)	–
Mixed race	2.7 (0–3.6)	1.14 (0.13–10.15)	–
Asian	3.1 (0–8.7)	1.51 (0.09–26.30)	–
Admission at public hospital			
No	1.1 (0.5–1.8)	1.00	1.00
Yes	4.5 (3.1–6.0)	6.52 (2.77–15.61)	5.07 (2.01–12.76)
Province			
Eastern Cape	4.7 (1.9–7.6)	1.00	
Free State	3.1 (0.9–6.1)	0.69 (0.17–2.78)	–
Gauteng	2.4 (0.8–4.0)	0.45 (0.15–1.75)	–
KwaZulu Natal	2.5 (0.8–4.2)	0.46 (0.15–1.45)	–
Western Cape	2.2 (0.1–3.4)	0.35 (0.08–1.10)	–
Other (Limpopo, Mpumalanga, North West Northern Cape)	1.6 (0.0–3.4)	0.32 (0.07–1.36)	–
Month			
March–May	2.0 (0.2–3.7)	1.00	–
June–July	2.8 (1.8–3.9)	1.45 (0.54–3.93)	–
August–September	2.3 (1.0–3.6)	1.11 (0.39–3.45)	–
≥1 non‐communicable underlying conditions^c^			
No	1.9 (1.2–2.6)	1.00	1.00
Yes	17.7 (8.2–27.2)	15.01 (5.16–38.06)	12.09 (4.19–34.89)
HIV infection			
No	2.4 (1.7–3.2)	1.00	–
Yes	6.6 (0.0–14.1)	2.68 (0.63–11.40)	–
Past tuberculosis			
Yes	2.6 (1.9–3.3)	1.00	–
No	2.1 (0.0–6.4)	0.71 (0.9–5.82)	–

Abbreviations: CFR, case fatality risk; CI, confidence interval; OR, odds ratio.

^a^ Individuals who were SARS‐CoV‐2 rRT‐PCR positive results AND were admitted to sentinel hospital AND had with complete outcome information, that is, either died or were discharged from hospital.

^b^ From univariable and multivariable models on imputed data. Multivariable model adjusted for age, birth sex, admission to public or private sectors, and presence of underlying conditions.

^c^ Included heart disease, diabetes, malignancy, renal disease, and obesity. In another model adding hypertension, chronic respiratory disease or asthma to definition of non‐communicable underlying medical condition; in addition to initial list (i.e. heart disease, diabetes, malignancy, renal disease and obesity), ≥1 non‐communicable underlying condition was still associated with mortality risk albeit with lower strength of association: aOR 3.29 (95% CI 1.19‐8.37).

## 4 DISCUSSION

In this study of pediatric COVID‐19 in South Africa, we found an overall 14.6% of children (0–17 years) tested using SARS‐CoV‐2 rRT‐PCR assays had positive tests, with positivity varying with age and province. The cumulative incidence of laboratory‐confirmed SARS‐CoV‐2 infection among children in our study was 228 per 100,000 children. The childhood in‐hospital CFR was 2.6%, lower than expected given elevated underlying vulnerabilities in the childhood population in the country. In‐hospital death was associated with age >10 years and infancy, male sex, and most strongly with having one or more reported underlying non‐communicable medical conditions.In our analysis, the overall SARS‐CoV‐2 rRT‐PCR testing rate among children was five times lower compared with the testing rate on adults. The percent test positive increased steadily with age suggesting increasing susceptibility, differences in clinical presentations or under‐ascertainment of infections with age. The SARS‐CoV‐2 rRT‐PCR percent test positive also varied by province from 9.9% in KwaZulu Natal, the province with the highest number of children in the country, to 17.2% in North West, the province with the lowest SARS‐CoV‐2 rRT‐PCR testing rate in the country. These provincial differences in test positivity and test rates could have been due to differences in burden of SARS‐CoV‐2 infections, differences in the implementation of the nationally recommended testing criteria, or in access to testing. In earlier weeks of the pandemic, the test positive rates were <10% but continued to increase over the course of the surveillance period, probably because of changes in testing criteria with later criteria limiting testing to individuals most likely to be positive—the symptomatic, those with underlying conditions or those requiring admissions.

The cumulative incidence of laboratory‐confirmed SARS‐CoV‐2 infection among South African children in our study was 228 per 100,000 children. This incidence was higher compared to rates of less than 100 per 100,000 children in Norway^35^ and Australia^36^ but lower than the 829 per 100,000 children in the United States in September 2020.^5^ These settings had higher test rates and lower proportion positive tests compared to South Africa, which means lower test rates alone may not explain the incidence of SARS‐CoV‐2 infection observed in South Africa. In our study, children made up less than 3.0% of SARS‐CoV‐2‐positive admissions and 0.4% of in‐hospital deaths at sentinel hospitals despite making up a third of the population; this confirms the global finding that COVID‐19 is less severe in children compared to adults.^37^ This similarity in the profile of childhood SARS‐CoV‐2 infections among children across different countries and provinces suggests presence of biological factors protective against severe disease in children. Earlier studies have suggested that (1) children's immune systems might have been trained by vaccinations or other viral infections resulting in robust innate immune responses upon reinfection with the same or unrelated organisms^38^, ^39^; (2) children might have lower but increasing density of angiotensin‐converting enzyme 2, the primary receptor target for SARS‐CoV‐2 virus in the respiratory tract^38^, ^39^; and (3) adults might experience immunosenescent cells as they advance in age, which could contribute to poor immune responses.^38^, ^39^ Additional research is required to better understand the physiology of this natural aging cell process and inform development of immunological interventions to prevent severe disease in the general population.

The in‐hospital CFR among children at all participating sentinel hospitals in our study was 2.6% overall and 4.7% among the subset of public sector hospitals. This was higher than the <1% that has been reported from studies of hospitalized children in China^40^ and Europe,^19^ similar to 2% from a study of hospitalized children in the United States,^21^ but lower than the 12% average reported in two Iranian studies of hospitalized children.^41^, ^42^ The childhood in‐hospital mortality in our study was also lower than the 11% reported in a study of hospitalized children from the Democratic Republic of Congo.^23^ This finding was notable because the children in our study had a similar age and sex distribution and lower prevalence of underlying medical conditions compared with other hospital studies (16% compared to 40–66% in other hospital studies).^13^, ^21^, ^22^ Consistent with other studies,^13^, ^22^, ^43^ higher in‐hospital CFR was associated with age <1 year. The CFR in those <1 year was four times higher compared to children aged 1‐4 years. The relatively higher risk of in‐hospital death independently associated with admission at public hospitals might reflect hospitalization of less severe cases in the private sector with parents seeking earlier care or providers being more cautious. It might also reflect limited access to treatment and earlier diagnosis and management of underlying medical conditions, low socio‐economic status, higher prevalence of risk factors associated with childhood mortality, and higher background child mortality in catchment areas resulting in more deaths unrelated to the SARS‐CoV‐2 infection in the public sector. However, it is not possible to definitively ascribe the cause of death to SARS‐COV‐2, as some children had terminal illnesses and were receiving palliative care for underlying conditions. Similar to other studies,^21^, ^44^, ^45^ we also found a very strong association between having underlying medical conditions and in‐hospital deaths. More research on causes of death and the contribution of underlying medical conditions to in‐hospital deaths among SARS‐CoV‐2 infected children admitted into hospitals is needed. In the interim, children with underlying conditions should be prioritized for testing and the provision of non‐pharmacological interventions strengthened for them.

Our study included a large number of children tested and admitted in both the private and public sectors. There was good coverage, including 100% of all private hospitals and close to 90% of the public hospitals. However, there were some limitations to our study. First, the data collection tools used in the surveillance were similar for both adults and children. The selection of underlying conditions did not address important underlying conditions common to children in our setting, for example, HIV exposure, prematurity, or malnutrition. Second, hospital staff collected data while providing routine service. There was missing clinical information in the data from both the laboratory and hospital surveillance systems. Key missing information included symptom(s) presence and duration, COVID 19 exposure history, indications for testing, indications for hospital admission, duration and severity of underlying conditions, and causes of death. For this reason, we were unable to adequately describe the clinical presentation among cases. Additionally, we likely underestimated the total burden of underlying conditions among admitted children with the differential effects overestimated or underestimated. Third, we were unable to review medical records to ascertain and fully classify causes of death among childhood SARS‐CoV‐2‐positive admissions. As we did not have clinical data or post‐mortem data, we were unable to determine whether the SARS‐CoV‐2 infection was co‐incidental or involved in the casual pathway to death. Fourth, the data on SARS‐CoV‐2‐positive admissions were collected from sentinel hospitals as opposed to all hospitals admitting individuals with SARS‐CoV‐2. We were therefore unable to calculate population level hospitalization rates. Fifth, we considered the first occurrence of hospitalization and may have missed deaths occurring on re‐admissions. Sixth, because our data collection forms were not designed to collect data on the multisystem inflammatory syndrome in children (MISC), we were unable to describe MISC which could contribute towards SARS‐CoV‐2 morbidity and mortality among children.^46^ Lastly, we were only able to describe mortality among hospitalized children. This meant that children who died outside the hospital were not included and we might have underestimated mortality associated with SARS‐CoV‐2 infection. Although there have not been reports of excess deaths among children during the surveillance period, an important proportion of deaths in children in our setting occurs at home, particularly in rural areas.^47^ Despite these limitations, our study provides new information on the spectrum and outcome of SARS‐CoV‐2 in children in an upper middle‐income country context. Despite a high incidence of childhood pneumonia, poverty, pollution exposure, TB, and HIV infection in our setting, the spectrum of pediatric disease was mild, and the number of deaths was relatively small. Further research to understand why most children do not develop severe disease, even in a middle‐income context, is needed and may provide important insights into new preventive or protective strategies.

## AUTHOR CONTRIBUTIONS

**Tendesayi Kufa:** Conceptualization; data curation; formal analysis; methodology. **Waasila Jassat:** Conceptualization; data curation; supervision. **Cheryl Cohen:** Conceptualization; methodology. **Stefano Tempia:** Formal analysis; methodology. **Maureen Masha:** Data curation. **Nicole Wolter:** Formal analysis. **Gillian Hunt:** Data curation. **Andronica Moipone Shonhiwa:** Formal analysis. **Joy Ebonwu:** Formal analysis. **Genevie M. Ntshoe:** Formal analysis. **Wellington Maruma:** Formal analysis. **Nomathamsanqa Ndhlovu:** Formal analysis. **Hlengani Mathema:** Formal analysis. **Liliwe Shuping:** Formal analysis. **Shabir Madhi:** Conceptualization. **Haroon Saloojee:** Conceptualization. **Heather J. Zar:** Conceptualization. **Lucille Blumberg:** Conceptualization.

## FUNDING

The study was supported by the National Department of Health, Republic of South Africa through core surveillance funding to the National Institute for Communicable Diseases.

## CONFLICT OF INTEREST

The authors have no conflict of interest to declare.

### PEER REVIEW

The peer review history for this article is available at https://publons.com/publon/10.1111/irv.12916.

## Supporting information


**Table S1:** Description of SARS‐CoV‐2 rRT‐PCR positive children <18 years in South Africa, 1 March 2020–19 September 2020 (N = 45 609)
Table S2: Description of SARS‐CoV‐2 rRT‐PCR positive hospital admissions among children <18 years in South Africa by province, 1 March 2020–19 September 2020 (N = 2007)
Table S3: Distribution of non‐missing variables among children with complete follow up and included in multivariable model (N = 1817)
Table S4: Factors associated with in‐hospital death among SARS‐CoV‐2 rRT‐PCR positive admissions in children <18 years, South Africa, 1 March 2020–19 September 2020

**Figure S1:** Number of SARS‐CoV‐2 rRT‐PCR tests^*^, percent positive tests and associated‐ hospital admissions among children <18 years by province and epidemiology week, South Africa, 1 March 2020–19 September 2020

## Data Availability

Data available on request due to privacy/ethical restrictions

## ENDNOTE

* See, for example, 45 C.F.R. part 46.102(l),^2^ 21 C.F.R. part 56; 42 U.S.C. §241(d); 5 U.S.C. §552a; 44 U.S.C. §3501 et seq.
