# Supplementary material for: Epidemiology of SARS‐CoV‐2 infection and SARS‐CoV‐2 positive hospital admissions among children in South Africa
Source: Influenza Other Respir Viruses. 2021 Nov 18;16(1):34–47. doi: 10.1111/irv.12916 (PMC9664941; doi:10.1111/irv.12916)
Supplement: Supplementary file 1 — Table S1: Description of SARS‐CoV‐2 rRT‐PCR positive children <18 years in South Africa, 1 March 2020–19 September 2020 (N = 45 609) Table S2: Description of SARS‐CoV‐2 rRT‐PCR positive hospital admissions among children <18 years in South Africa by province, 1 March 2020–19 September 2020 (N = 2007) Table S3: Distribution of non‐missing variables among children with complete follow up and included in multivariable model (N = 1817) Table S4: Factors associated with in‐hospital death among SARS‐CoV‐2 rRT‐PCR positive admissions in children <18 years, South Africa, 1 March 2020–19 September 2020 [file IRV-16-34-s001.docx]

**Table S1: Description of SARS-CoV-2 rRT-PCR positive children <18 years in South Africa, 1 March 2020 -19 September 2020 (N= 45,609)**

| **Characteristic** | **All children, N= 45,609** |
| --- | --- |
| Age (median, IQR) | 12.4 (7.5 -15.7) |
| *Age in years, n (%)* |  |
| <1 | 2,232 (4.9) |
| 1 | 1,500 (3.3) |
| 2 | 1,200 (2.6) |
| 3 | 1,186 (2.6) |
| 4 | 1,358 (3.0) |
| 5 | 1,497 (3.3) |
| 6 | 1,620 (3.6) |
| 7 | 1,802 (4.0) |
| 8 | 1,969 (4.3) |
| 9 | 2,073 (4.6) |
| 10 | 2,347 (5.2) |
| 11 | 2,683 (5.9) |
| 12 | 3,453 (7.6) |
| 13 | 3,484 (7.6) |
| 14 | 3,398 (7.5) |
| 15 | 3,644 (8.0) |
| 16 | 4,120 (9.0) |
| 17 | 6,043 (13.3) |
| *Sex* |  |
| Female | 24,308 (53.3) |
| Male | 20,412 (44.8) |
| Missing | 889 (2.0) |
| *Testing laboratory, n (%)** |  |
| Private | 23,978 (52.6) |
| Public | 21,631 (47.4) |

rRT-PCR = real-time reverse transcriptase polymerase chain reaction; IQR= interquartile range; * refers to whether the laboratory where test was conducted was operated by the government or operated by a private entity

**Table S2: Description of SARS-CoV-2 rRT-PCR positive hospital admissions among children <18 years in South Africa by province, 1 March 2020 -19 September 2020 (N= 2,007)**

| **Variable** | **Eastern Cape** | **Free State** | **Gauteng** | **KwaZulu-Natal** | **Limpopo** | **Mpumalanga** | **North West** | **Northern Cape** | **Western Cape** | **South Africa** |
| --- | --- | --- | --- | --- | --- | --- | --- | --- | --- | --- |
|  | **(N=225)** | **(N=137)** | **(N=413)** | **(N=360)** | **(N=58)** | **(N=60)** | **(N=81)** | **(N=44)** | **(N=629)** | **(N=2007)** |
| ***Sex*** | | | | | | | | | | |
| Male, n (%) | 94 (41.8) | 69 (50.4) | 202 (49.2) | 184 (51.1) | 33 (56.9) | 28 (46.7) | 37 (45.7) | 22 (50.0) | 335 (53.3) | 1006 (50.1) |
| ***Median Age*** | | | | | | | | | | |
| Years, Median (IQR) | 13.9 (4.0-17.0) | 11.9 (2.8-15.8) | 6.7 (1.2.-13.7) | 7.6 (1.2-14.2) | 8.5 (1.1-15.4) | 6.1 (1.1-15.5) | 13.9 (4.3-16.8) | 9.0 (2.9-13.5) | 2.5 (0.5-10.4) | 6.8 (1.1-14.3) |
| ***Age Group*** | | | | | | | | | | |
| <1 year, n (%) | 28 (12.4) | 14 (10.2) | 94 (22.8) | 82 (22.8) | 14(24.1) | 13 (21.7) | 9 (11.1) | 5 (11.4) | 210 (33.4) | 469 (23.4) |
| 1-4 years, n (%) | 32 (14.2) | 32 (23.4) | 94 (22.8) | 70 (19.4) | 5 (8.6) | 16 (26.7) | 15 (18.5) | 10 (22.7) | 175 (27.8) | 449 (22.4) |
| 5-9 years, n (%) | 24 (10.7) | 15 (11.0) | 63 (15.3) | 50 (13.9) | 12 (20.7) | 6 (10.0) | 8 (9.9) | 8 (18.2) | 81 (12.9) | 267 (13.3) |
| 10-14 years, n (%) | 44 (19.6) | 31 (22.6) | 82 (19.9) | 83 (23.1) | 11 (19.0) | 8 (13.3.) | 15 (18.5) | 14 (31.8) | 78 (12.4) | 366 (18.2) |
| 15-17 years, n (%) | 97 (43.1) | 45 (32.9) | 80 (19.4) | 75 (20.8) | 16 (27.6) | 17 (28.3) | 34 (42.0) | 7 (15.9) | 85 (13.5) | 456 (22.7) |
| ***Hospital**** | | | | | | | | | | |
| Public, n (%) | 181 (80.4) | 66 (48.2) | 28 (6.8) | 89 (24.7) | 30 (51.7) | 5 (8.3) | 24 (29.6) | 2 (4.6) | 465 (73.9) | 890 (44.3) |
| ***Underlying conditions*** | | | | | | | | | | |
| Data available (Yes), n (%) | 196 (87.1) | 134 (97.8) | 346 (83.8) | 278 (77.2) | 57 (98.3) | 51 (85.0) | 74 (91.4) | 31 (70.5) | 259 (41.2) | 1,426 (71.1) |
| Has ≥1 underlying conditions reported**, n (%) | 16 (8.2) | 14 (10.6) | 25 (7.3) | 27 (9.7) | 2 (3.5) | 4 (7.8) | 4 (5.4) | 2 (6.5) | 137 (55.2) | 231 (16.2) |
| ***Severity of disease*** | | | | | | | | | | |
| LOS, days (median, IQR) | 6 (3-11) | 3 (1-7) | 3 (1-6) | 4 (2-7) | 3.5 (2-5) | 2 (1-5) | 4 (2-7) | 2 (1-3) | 3 (1.6) | 3 (2-7) |
| Ever admitted to high care , n (%) | 5 (2.2) | 5 (3.7) | 36 (8.7) | 17 (4.7) | 1 (1.7) | 8 (13.3) | 2 (2.5) | 3 (6.8) | 7 (1.1) | 84 (4.2) |
| Ever admitted ICU, n (%) | 9 (4.0) | 3 (2.2) | 50 (12.1) | 27 (7.5) | 4 (6.9) | 7 (11.7) | 2 (2.5) | 1 (2.3) | 51 (8.1) | 154 (7.7) |
| Ever ventilated, n (%) | 8 (3.6) | 3 (2.2) | 19 (4.6) | 13 (3.6) | 4 (6.9) | 2 (3.3) | 1 (1.2) | 1 (2.3) | 6 (1.0) | 57 (2.8) |
| ***Outcomes*** | | | | | | | | | | |
| Discharged alive, n (%) | 200 (88.9) | 126 (92.0) | 361 (87.0) | 316 (87.8) | 36 (62.1) | 54 (90.0) | 55 (67.9) | 39 (88.6) | 582 (92.5) | 1,769 (87.9) |
| Transferred out, n (%) | 3 (1.3) | 0 (0.0) | 1 (0.2) | 9 (2.5) | 0 (0.0) | 0 (0.0) | 2 (2.5) | 0 (0.0) | 1 (0.2) | 16 (0.8) |
| Still admitted, n (%) | 12 (5.3) | 7 (5.1) | 42 (10.2) | 27 (7.5) | 21 (36.2) | 4 (6.7) | 24 (29.6) | 5 (11.4) | 32 (5.1) | 174 (8.7) |
| Died, n (%) | 10 (4.4) | 4 (2.9) | 9 (2.2) | 8 (2.2) | 1 (1.7) | 2 (3.3) | 0 (0.0) | 0 (0.0) | 13 (2.1)** | 47 (2.3) |

ICU= intensive care unit; IQR= interquartile range, LOS= length of stay * refers to whether hospital was operated by the government or by a private entity; excludes one child who died from a cause deemed not SARS-CoV-2 related.

**Table S3: Distribution of non-missing variables among children with complete follow up and included in multivariable model (N=1,817)**

| **Variable** | **Non-missing (n, %)** |
| --- | --- |
| Age | 1,817 (100) |
| Birth sex | 1,817 (100) |
| Province | 1,817 (100) |
| Month of admission | 1,814 (99.8) |
| Ethnicity (race) | 1,358 (74.7) |
| TB | 1,305 (71.8) |
| Malignancy | 1,305 (71.8) |
| Diabetes | 1,305 (71.8) |
| Hypertension | 1,305 (71.8) |
| Asthma/ chronic pulmonary disease | 1,305 (71.8) |
| HIV | 1,220 (67.1) |
| Obesity | 1,141 (61.0) |

**Table S4: Factors associated with in-hospital death among SARS-CoV-2 rRT-PCR positive admissions in children <18 years, South Africa, 1 March 2020 – 19 September 2020**

| **Variable** | **n/N (%)** | **Univariate OR (95% CI )^µ^** | **Multivariate OR (95% CI )^µ^**  **N=1305*** |
| --- | --- | --- | --- |
| *Age in years* |  |  |  |
| <1 years | 16/425 (3.5) | 4.22 (1.18- 15.18) | 4.15 (1.01 – 17.07) |
| 1-4years | 3/412 (0.7) | 1.00 | 1.00 |
| 5-9 years | 5/242 (2.1) | 2.85 (0.66- 12.25) | 1.12 (0.20- 6.32) |
| 10- 14 years | 13/327 (3.4) | 4.92 (1.33- 18.14) | 3.82 (0.95- 15.32) |
| ≥15 years | 15/411 (3.7) | 5.65 (1.58- 20.28 | 3.40 (0.83- 13.88) |
| *Male* |  |  |  |
| No | 17/894 (2.1) | 1.00 | 1.00 |
| Yes | 34/925 (3.7) | 1.70 (0.92- 3.15) | 1.78 (0.81- 3.91) |
| *Ethnicity* |  |  |  |
| White | 1/55 (1.8) | 1.00 | -- |
| Black African | 35/1144 (3.1) | 1.77 (0.22- 13.90) | -- |
| Mixed race | 3/120 (2.5) | 1.51 (0.14- 16.07) | -- |
| Asian | 1/36 (2.8) | 1.64 (0.09- 29.41) | -- |
| *Admission at public hospital* |  |  |  |
| No | 12/1,044 (1.2) | 1.00 | 1.00 |
| Yes | 41/779 (5.3) | 5.46 (2.37 -12.56) | 8.79 (3.12- 24.75) |
| *Province*** |  |  |  |
| Eastern Cape | 10/212 (4.7) | 1.00 | -- |
| Free State | 4/130 (3.1) | 0.69 (0.17- 2.78) | -- |
| Gauteng | 13/375 (3.5) | 0.45 (0.15- 1.35) | -- |
| KwaZulu Natal | 10/324 (3.1) | 0.46 (0.15- 1.45) | -- |
| Western Cape | 13/595 (2.2) | 0.35 (0.11- 1.10) | -- |
| Other (Limpopo , Mpumalanga , North West , Northern Cape) | 3/187 (1.6) | 0.32 (0.05- 1.36) | -- |
| *Month* |  |  |  |
| March- May 2020 | 5/249 (2.0) | 1.00 | -- |
| June- July 2020 | 30/1,052 (2.9) | 1.46 (0.54- 3.93) | -- |
| August- September 2020 | 12/513 (2.3) | 1.16 (0.39- 3.45) | -- |
| *≥1 underlying condition**** |  |  |  |
| No | 9/685 (1.3) | 1.00 | 1.00 |
| Yes | 27/620 (4.4) | 4.13 (1.73- 9.85) | 4.27 (1.78- 10.26) |
| *HIV* |  |  |  |
| No | 25/191 (2.1) | 1.00 | -- |
| Yes | 2/29 (6.9) | 3.11 (0.61- 15.89) | -- |
| *Previous TB* |  |  |  |
| No | 35/1227 (2.7) | 1.00 | -- |
| Yes | 1/28 (3.6) | 1.04 (0.11- 10.00) | -- |

^µ^- models run on un-imputed data. Multivariable model included age, birth sex, admission at public hospital and having one or more underlying conditions; CI= confidence interval; OR = odds ratio; *individuals with complete outcome information; *** included heart disease, diabetes, malignancy, renal disease and obesity.
