# Supplementary material for: Epidemiology of SARS‐CoV‐2 infection and SARS‐CoV‐2 positive hospital admissions among children in South Africa
Source: Influenza Other Respir Viruses. 2021 Nov 18;16(1):34–47. doi: 10.1111/irv.12916 (PMC9664941; doi:10.1111/irv.12916)
Supplement: Supplementary file 2 — Figure S1: Number of SARS‐CoV‐2 rRT‐PCR tests*, percent positive tests and associated‐ hospital admissions among children <18 years by province and epidemiology week, South Africa, 1 March 2020–19 September 2020 [file IRV-16-34-s002.docx]

**Supplementary Figure S1: Number of SARS-CoV-2 rRT-PCR tests^*^, percent positive tests and associated- hospital admissions among children <18 years by province and epidemiology week, South Africa, 1 March 2020 – 19 September 2020**
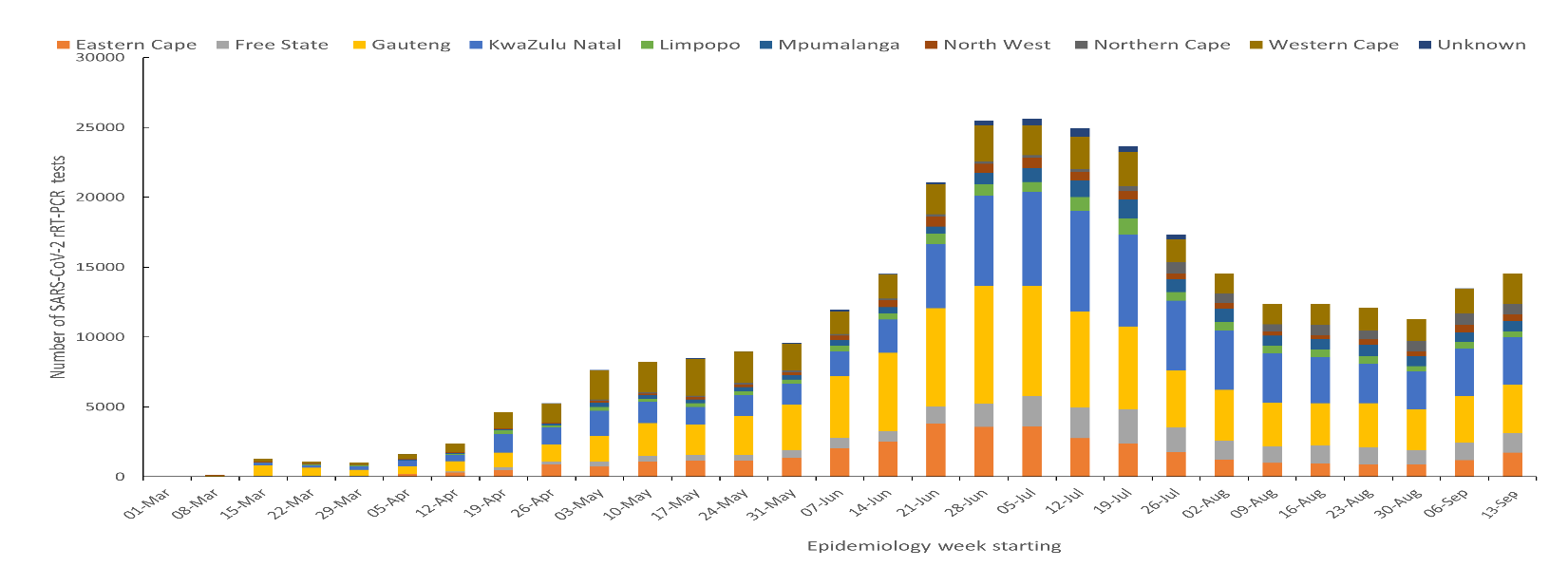


Fig S1a


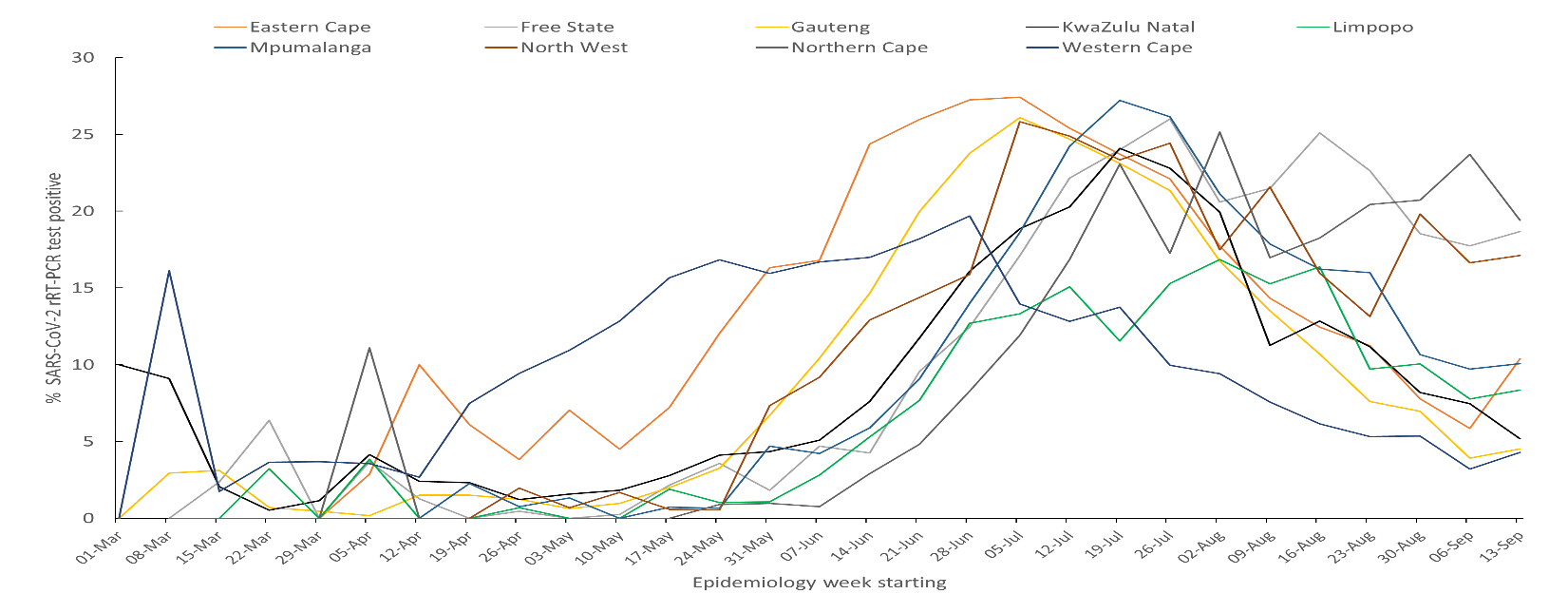


Fig S1b


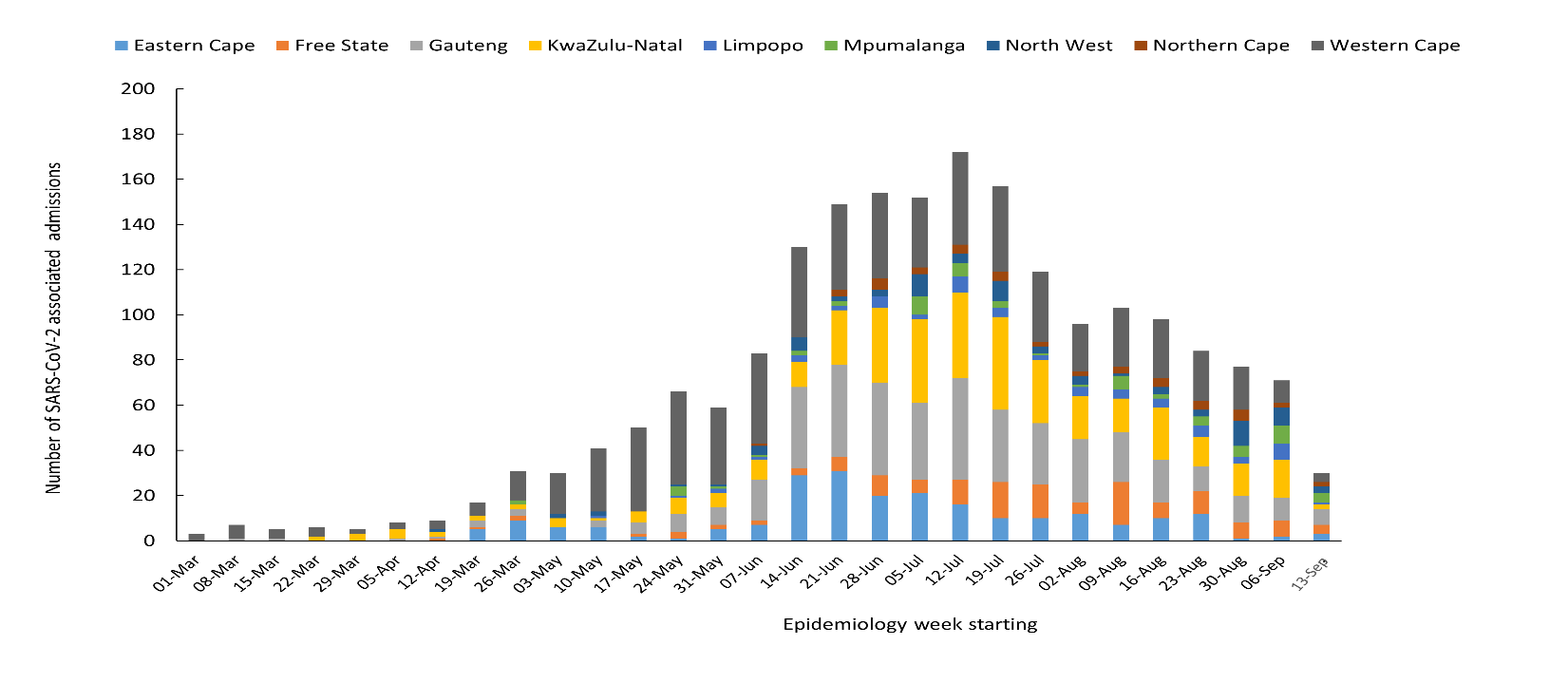


Fig S1c

rRT-PCR= real-time reverse transcriptase polymerase chain reaction
